# Development and validation of an AI use scale for sport and exercise science students

**DOI:** 10.1038/s41598-026-45316-4

**Published:** 2026-03-21

**Authors:** Zhihai He, Xingmin Han, Yang Ruizhu, Li Li

**Affiliations:** 1 Department of Physical Education, Harbin Sport University, Harbin, Heilongjiang Province China; 2 Institute for Health and Sport (IHES), Victoria University, Melbourne, Australia; 3 Department of Physical Education, Shaanxi Xueqian Normal University, Xi’an, Shaanxi Province China

**Keywords:** Artificial Intelligence, AI Literacy, Sport and Exercise Science, Questionnaire Development, Psychometric Validation, Ethics and Disclosure, Curriculum Design, Higher Education, Mathematics and computing, Psychology, Psychology

## Abstract

Artificial intelligence (AI) is rapidly transforming sport and exercise domains. Yet, sport-science curricula have lagged in integrating AI literacy, and little is known about students’ knowledge, ethical practices, and perceptions regarding AI. Existing measurement tools are often generic and ill-suited to sport education contexts. This study aimed to develop and validate a concise, domain-specific questionnaire to assess sport students’ AI use. A systematic instrument development process was used, including literature review, expert consultation, item generation, and psychometric validation. The resulting 14-item tool covers four domains: AI Awareness, Ethics & Disclosure, Trust & Verification, and Course & Institution Expectations. The instrument was administered to 864 undergraduate sport-science students in China and analysed using exploratory factor analysis (EFA) and confirmatory factor analysis (CFA) on separate training and test sets. EFA supported a four-factor structure with high sampling adequacy (KMO = 0.95) and strong communalities. CFA confirmed good model fit (CFI = 1.00, TLI = 0.99, RMSEA = 0.09, SRMR = 0.05). Subscales demonstrated excellent internal consistency (Cronbach’s α = 0.90–0.94; McDonald’s ω = 0.90–0.94), convergent validity (AVE = 0.77–0.87), and discriminant validity (HTMT ratios < 0.85). This validated, context-specific instrument provides educators with a reliable tool to assess and enhance AI literacy in sport education. The findings support the integration of targeted AI training, ethical instruction, and institutional policies to prepare students for responsible, real-world AI engagement in sport and health domains.

**Supplementary Information:**

The online version contains supplementary material available at 10.1038/s41598-026-45316-4.

## Introduction

Artificial intelligence (AI) technologies are now widely deployed across the sport domain^1^. In performance analytics and optimisation, AI converts high-volume physiological and match data into athlete-specific insights, enabling precise training adjustments and lowering injury risk^2^. Machine-learning models of load and movement estimate injury probability and flag early risks^3,4^. For program design, AI generates personalised plans that adapt to individual needs and real-time feedback^5,6^. In education, computer vision and wearables capture movement and fitness data, producing objective, fine-grained metrics for fair, data-driven assessment^7,8^. Given AI’s growing importance in sport, it is necessary to integrate AI into university curricula for sport students so that students receive training that combines theory with practice and graduate with competence in data analysis and AI applications.

Despite ongoing advances, we still lack a systematic understanding of how future sport professionals, sport and exercise science students, engage with AI^9^. Their acceptance of AI tools, specific usage scenarios and purposes, levels of knowledge and skill, and baseline practices in ethics and compliance are rarely reported. Addressing this evidence gap is essential for designing and evaluating university AI curricula tailored to sport. Such data would also underpin academic-integrity and risk-governance frameworks (e.g., clearly defined permitted/prohibited use cases, disclosure templates, detection and appeal processes) and enable tiered risk management. In addition, they would inform staff development and resource allocation by identifying training deficits, prioritising modules such as prompt engineering, data anonymisation, and bias detection, and optimising software licences and computing provision.

Several instruments have been used to survey university students’ AI use or AI literacy, however, most have been developed for general educational contexts and broad student populations^10–13^. As a result, some items focus primarily on conceptual knowledge of machine-learning principles or the technical mechanisms of large language models^13^. In sport and exercise science education, however, students may encounter AI in more applied scenarios, such as training analysis, athlete monitoring, and rehabilitation planning. Consequently, certain domain-specific applications of AI may not be fully captured by existing general-purpose instruments.

In addition, existing scales are frequently lengthy (often dozens of items), which limits their practicality^13^. Furthermore, these instruments rarely assess students’ understanding of course or institutional expectations regarding appropriate AI use in academic contexts^13^. Together, these observations suggest that there may be value in developing a concise instrument designed to capture AI-related awareness, practices, and expectations within sport and exercise science education.

In response, this study aims to develop a concise questionnaire specifically for sport and exercise science students to assess their AI-related knowledge, use, and needs. The instrument is tailored to the distinctive context of sport education and encompasses four domains: (i) AI Awareness: foundational understanding of AI; (ii) Ethics & Disclosure: honest reporting and prior disclosure; (iii) Trust & Verification: seek sources, check bias, use outputs cautiously; and (iv) Course & Institution Expectations: practical training and clear rules for use and disclosure. Items are contextualised with examples from athlete-monitoring, coaching workflows, and training environments to maximise relevance and interpretability. The scale is deliberately streamlined to balance brevity with adequate content coverage. Its primary goal is to characterise students’ current understanding and patterns of use, identify gaps and misconceptions, and elicit educational needs and expectations. Findings from this survey will have clear practical value. They can guide curriculum development and pedagogy (e.g., updating courses to include hands-on AI training), inform targeted workshops and student resources, and help educators gauge students’ readiness to adopt AI within sport-science practice.

## Methods

### Study design and overall procedure

We followed a systematic instrument-development workflow comprising literature review, item generation and construct definition, expert review, pilot testing, and psychometric validation (reliability and validity).

## Literature review

Between August and October 2025, we searched Web of Science Core Collection, MEDLINE (Ovid), Embase (Ovid), and PsycINFO using combinations of the keywords AI, large language model, university students, AI literacy, and AI use. To capture grey literature, we additionally searched Google Scholar and major preprint servers.

### Item generation and construct definition

Item generation and construct specification were further guided by a review of conceptually related instruments^13–24^. Thirty candidate items were drafted initially. After multiple rounds of discussion, four core constructs were retained: AI Awareness (AW; 4 items), Ethics & Disclosure (ED; 3 items), Trust & Verification (TV; 5 items), and Course & Institution Expectations (CIE; 4 items), for a total of 16 items. All items used a 5-point Likert scale (1 = strongly disagree, 5 = strongly agree).

To describe students’ AI use profiles, we added Module A (Usage; 6 items, not scored) covering frequency, purposes, modality types, and prior training. All experts agreed that the resulting blueprint adequately covered the target constructs and context for sports students.

### Expert review and content revision

To ensure content validity and semantic clarity, three additional experts (education, sport science, and artificial intelligence) independently reviewed the item pool. Reviewers evaluated item wording, conceptual coverage, and cultural appropriateness. Based on their feedback, we removed semantically overlapping or ambiguous items and refined technical terms to better match the discourse of university sports students.

### Online survey construction and administration

The revised instrument was migrated to Qualtrics for electronic administration. The survey implemented forced-response settings and skip/branch logic, and underwent multiple rounds of testing on mobile and desktop to ensure UI compatibility and complete data export. A technical dry run was conducted prior to formal launch.

### Psychometric validation

#### Participants

Participants were undergraduate students (Years 1–4) enrolled in the Physical Education program at a university in northern China. The instrument was distributed to 1,000 students in the same program. These participants were randomly allocated to a training set (70%) and a test set (30%). Descriptive statistics were used to summarize baseline characteristics.

### Pilot testing and revision

Forty target participants (not part of the analytic sample) completed the test and provided feedback on the questionnaire’s structure, item comprehensibility, and the clarity of the response-scale anchors. The team then adjusted item order and streamlined wording in response to the feedback.

### Exploratory factor analysis

For exploratory factor analysis (EFA) we used the training set. Because items were ordinal Likert-type, we estimated a polychoric correlation matrix; when needed, the matrix was smoothed to near–positive definiteness. Sampling adequacy was evaluated using the Kaiser–Meyer–Olkin (KMO) index and Bartlett’s test of sphericity. Factors were extracted with minimum residuals (minres) and rotated obliquely (oblimin) to allow correlated factors. The number of factors was determined a priori by theory and empirically by parallel analysis (1,000 random permutations). Items were retained if they met all pre-specified criteria: primary loading ≥ 0.40, largest cross-loading < 0.30, and communality (h²) ≥ 0.30. Items failing any criterion were removed and the EFA was re-estimated on the trimmed set.

### Confirmatory factor analysis

A confirmatory factor analysis (CFA) was then fit to the test set to evaluate the factor structure identified by EFA. CFA treated items as ordered categorical and was estimated with robust weighted least squares. Model fit was assessed using χ²/df (Chi-square divided by degrees of freedom), CFI (Comparative Fit Index), TLI (Tucker-Lewis Index), RMSEA (Root Mean Square Error of Approximation), and SRMR (Standardized Root Mean Square Residual), interpreted against conventional cut-offs (χ²/df < 5, CFI/TLI ≥ 0.95, RMSEA ≤ 0.08–0.10, SRMR ≤ 0.08). Standardized factor loadings and latent factor correlations were reported.

Internal consistency was evaluated for each subscale in the training set, test set, and full sample using Cronbach’s α and McDonald’s ω (Cronbach alpha ≥ 0.8, McDonald’s omega ≥ 0.7 indicating adequacy). Convergent validities were quantified using Average Variance Extracted (AVE) (AVE ≥ 0.50 indicating adequacy). Discriminant validity was assessed using the heterotrait–monotrait (HTMT) ratio (HTMT ratio ≤ 0.85 indicating adequacy). All analyses were conducted in R.

### Ethics and informed consent

Institutional ethics approval was obtained prior to data collection. All methods were performed in accordance with the relevant guidelines and regulations, including the Declaration of Helsinki. The survey landing page described the study purpose, anonymity principles, data storage, and the right to withdraw. Participants provided electronic informed consent before proceeding. All data were used solely for academic research and not for any other purposes without authorization. The development and administration of the instrument adhered to ethical standards for social-science research and aligned with procedures commonly used in studies of AI use.

## Results

### Demographic variables

A thousand participants were invited. By October 8, 2025, 864 responses were received. (response rate = 86.4%). All responses were complete. Respondents were randomly allocated in a 70:30 ratio to a training set (*n* = 604) and a test set (*n* = 260). Table 1 summarizes the baseline characteristics of two sets. Overall, the two sets showed similar distributions across demographic and comparable.

**Table 1:** Baseline characteristics of the training and test samples.

Variable	Level	Train (*n* = 604)	Test (*n* = 260)
Parental education level, n (%)	Bachelor’s degree or above	94 (15.6%)	37 (14.2%)
	Diploma/Certificate	201 (33.3%)	94 (36.2%)
	Year 12 or below	309 (51.2%)	129 (49.6%)
Sex, n (%)	Male	475 (78.6%)	221 (85.0%)
	Female	129 (21.4%)	39 (15.0%)
Grade, n (%)	Grade 1	152 (25.2%)	79 (30.4%)
	Grade 2	45 (7.5%)	22 (8.5%)
	Grade 3	252 (41.7%)	87 (33.5%)
	Grade 4	155 (25.7%)	72 (27.7%)
Only child, n (%)	No	353 (58.4%)	151 (58.1%)
	Yes	251 (41.6%)	109 (41.9%)
Living area, n (%)	Urban	228 (37.7%)	85 (32.7%)
	Rural	376 (62.3%)	175 (67.3%)
Body Mass Index (kg/m^2)	Mean ± SD	23.3 ± 6.5	23.2 ± 5.8

Descriptive results from Module A provide contextual information about students’ AI use patterns (Table 2). Most participants reported having used AI tools (84.14%). In terms of frequency, over half of the students indicated using AI 1–2 times in everyday life (53.59%), while approximately one third reported using AI three or more times (37.61%). The most commonly reported purposes for AI use were coursework writing or summarization (70.37%) and information searching (60.19%). Around one quarter of students reported using AI for planning and scheduling (26.27%) or training and rehabilitation-related tasks (28.82%).

The descriptive statistics of the 16 items are also presented in Table 2. Overall, item means ranged from 3.22 to 3.56, indicating generally moderate to relatively high levels of agreement among participants. Items within the Ethics & Disclosure and Course & Institution Expectations domains showed slightly higher average scores.

**Table 2:** **Descriptive statistics of the items in the AI Use Scale for Sport and Exercise Science Students**.

Factor	Item	Level	All samples (864)
Module A — AI Usage	QA1	No	137 (15.86%)
		Yes	727 (84.14%)
	QA2	Never	76 (8.80%)
		1–2 times	463 (53.59%)
		3–4 times	228 (26.39%)
		Almost every day	79 (9.14%)
		Multiple times per day	18 (2.08%)
	QA3	Communication & email	162 (18.75%)
		Coursework writing/summary	608 (70.37%)
		Creative work (image/audio/video)	250 (28.94%)
		Data/code	139 (16.09%)
		Information search	520 (60.19%)
		Planning & scheduling	227 (26.27%)
		Training/rehabilitation-related	249 (28.82%)
		Other	70 (8.10%)
	QA4	No	699 (80.90%)
		Yes	165 (19.10%)
AI Awareness (AW)	QAW1	Mean ± SD	3.36 ± 0.82
	QAW2	Mean ± SD	3.22 ± 0.82
	QAW3	Mean ± SD	3.23 ± 0.82
	QAW4	Mean ± SD	3.28 ± 0.79
Ethics & Disclosure (ED)	QB1	Mean ± SD	3.44 ± 0.82
	QB2	Mean ± SD	3.47 ± 0.80
	QB3	Mean ± SD	3.56 ± 0.82
Trust & Verification (TV)	QC1	Mean ± SD	3.53 ± 0.81
	QC2	Mean ± SD	3.45 ± 0.82
	QC3	Mean ± SD	3.49 ± 0.83
	QC4	Mean ± SD	3.48 ± 0.78
	QC5	Mean ± SD	3.51 ± 0.77
Course & Institution Expectations (CIE)	QE1	Mean ± SD	3.45 ± 0.77
	QE2	Mean ± SD	3.53 ± 0.75
	QE3	Mean ± SD	3.54 ± 0.76
	QE4	Mean ± SD	3.46 ± 0.78

Note: Items marked with * were reverse-scored before analysis.

### Exploratory factor analysis

EFA was conducted on the training set. Sampling adequacy and sphericity were assessed. The KMO index (KMO = 0.95)^25^, and Bartlett’s test of sphericity was significant (*p* < 0.001), all indicated excellent adequacy. Item-level measures of sampling adequacy were all ≥ 0.84 (Table S1, Supplementary File 1). These diagnostics support the factorability of the correlation matrix and the suitability of the data for subsequent EFA/CFA.

EFA used a polychoric correlation matrix (smoothed and adjusted to near–positive definiteness), minres extraction, and oblimin oblique rotation. The number of factors was set to four based on parallel analysis and theoretical expectations (Table S2). Items were retained if the primary loading ≥ 0.40, the largest cross-loading < 0.30, and communality (h²) ≥ 0.30. In total, 14 items were retained; QC1 and QC3 were removed due to cross-loadings exceeding the threshold (maximum secondary loadings 0.33, see Table S3, Supplementary File 1).

Table 3 presents the trimmed four-factor solution obtained after removing items that failed the retention criteria in the EFA. Based on item content and loading patterns, the factors were labelled AI Awareness (AW; QAW1–QAW4), Ethics & Disclosure (ED; QB1–QB3), Trust & Verification (TV; QC2, QC4, QC5), and Course & Institution Expectations (CIE; QE1–QE4). Primary loadings ranged 0.55–0.91 for CIE, 0.52–0.93 for AW, 0.72–0.89 for TV, and 0.63–0.78 for ED. Item communalities (h²) were mostly 0.71–0.94, indicating strong association between items and their intended factors. All eigenvalues exceeded 1 (AW = 2.93, ED = 2.10, TV = 2.72, CIE = 3.21), accounting for 26.72%, 19.18%, 24.82%, and 29.27% of the variance, respectively, supporting a coherent four-factor structure and satisfactory measurement quality. The final 14-item AI use scale (after item removal) can be found in Supplemental file 2, Table S2.

**Table 3:** Exploratory factor analysis results.

Item	Latent factors				
	AI Awareness	Ethics & Disclosure	Trust & Verification	Course & Institution Expectations	h²
QAW3	0.93				0.85
QAW4	0.87				0.81
QAW2	0.84				0.77
QAW1	0.52				0.53
QB2		0.78			0.8
QB3		0.64			0.82
QB1		0.63			0.72
QC4			0.89		0.85
QC2			0.84		0.74
QC5			0.72		0.83
QE2				0.91	0.94
QE3				0.86	0.87
QE1				0.82	0.72
QE4				0.55	0.71
Eigenvalues	2.93	2.1	2.72	3.21	
Variance explained (%)	26.72%	19.18%	24.82%	29.27%	

### Confirmatory factor analysis

**Fig. 1:**
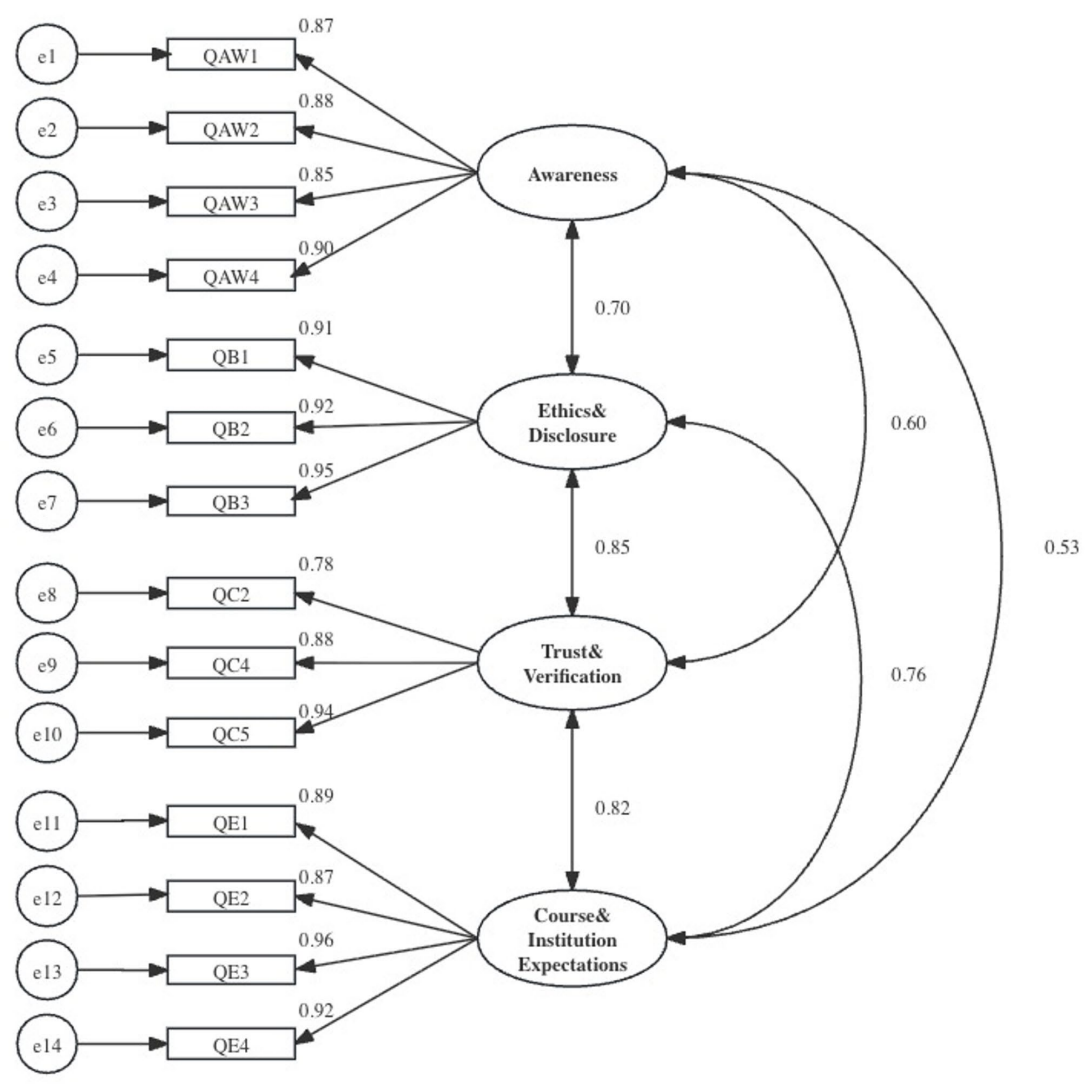
Confirmatory factor analysis of scale.

CFA on the validation set evaluated the 14-item scale. Standardized loadings ranged from 0.78 to 0.97. Latent factor correlations ranged from 0.53 to 0.85, indicating related yet distinguishable constructs (Fig. 1; Tables S3–S4, Supplementary File 1). Model-fit indices are reported in Table 4, indicating overall fair-to-excellent fit.

**Table 4:** CFA model fit indices.

X2/df	Perfect fit values	Good fit values	Values	Fit level
	≤ 3	3– 5	3.20	Good
CFI	≥ 0.95	0.90–0.95	1.00	Perfect
TLI	≥ 0.95	0.90–0.95	0.99	Perfect
RMSEA	0.00–0.05	0.05–0.10	0.09	Good
SRMR	0.00–0.05	0.05–0.10	0.05	Perfect

Note: χ²/df: Chi-square divided by degrees of freedom, CFI: Comparative Fit Index, TLI: Tucker-Lewis Index, RMSEA: Root Mean Square Error of Approximation, SRMR: Standardized Root Mean Square Residual.

### Internal consistency

Cronbach’s α and McDonald’s ω results are reported in Table 5. The coefficients ranged from 0.90 to 0.94 across different sample splits. These results indicate excellent internal consistency for all four latent variables in the training set, test set, and full sample.

**Table 5:** Cronbach alpha and McDonald’s omega reliability coefficient results.

Latent factors	Cronbach alpha			McDonald’s omega		
	Train	Test	Full	Train	Test	Full
AI Awareness	0.91	0.92	0.91	0.91	0.92	0.91
Ethics & Disclosure	0.90	0.94	0.92	0.90	0.94	0.92
Trust & Verification	0.92	0.90	0.91	0.92	0.90	0.92
Course & Institution Expectations	0.94	0.94	0.94	0.94	0.94	0.94

Note: Cronbach alpha ≥ 0.8, McDonald’s omega ≥ 0.7 indicates adequate internal consistency.

### Discriminant validity

HTMT values ranged from 0.50 to 0.82 across construct pairs, all below the conservative 0.85 threshold, indicating that the constructs are statistically distinguishable (Table 6). Correlations between latent variables were lower than 0.85, also indicated a good discriminant validity (Table S5, supplemental file 1).

**Table 6:** Heterotrait–Monotrait Ratio Matrix for Discriminant Validity.

Latent factors	AI Awareness	Ethics & Disclosure	Trust & Verification	Course & Institution Expectations
AI Awareness	1.00	0.70	0.59	0.50
Ethics & Disclosure	0.70	1.00	0.82	0.72
Trust & Verification	0.59	0.82	1.00	0.82
Course & Institution Expectations	0.50	0.72	0.82	1.00

Note: HTMT ratio ≤ 0.85 indicates adequate discriminant validity.

### Convergent validity

As shown is Table 7, AVEs ranged from 0.77 to 0.87, exceeding conventional benchmarks (AVE ≥ 0.50), indicating stable item performance and substantial variance explained by each construct.

**Table 7:** Average Variance Extracted of Latent Factors.

Latent factors	Average variance extracted (AVE)
AI Awareness	0.77
Ethics & Disclosure	0.87
Trust & Verification	0.77
Course & Institution Expectations	0.84

Note: AVE ≥ 0.50 indicates satisfactory convergent validity.

## Discussion

This study developed and validated a concise questionnaire to assess sport and exercise science students’ knowledge, use, and needs related to AI. Guided by theoretical and contextual considerations, the instrument was structured around four domains – AI Awareness, Ethics & Disclosure, Trust & Verification, and Course & Institution Expectations. Exploratory and confirmatory factor analyses supported a coherent four-factor structure with strong psychometric properties, indicating that the developed tool reliably captures distinct but interrelated dimensions of AI literacy among students in sport-related disciplines.

The four-factor solution aligns closely with both theoretical expectations and current discourses surrounding responsible AI use in education^26^. The AI Awareness factor reflects foundational understanding and awareness of AI applications and limitations, echoing calls to embed digital literacy and AI competence into higher education curricula^27^. The Ethics & Disclosure factor represents students’ integrity and transparency when using AI tools – issues that have gained increasing attention in academic integrity frameworks. The Trust & Verification factor captures the ability to critically assess AI-generated outputs, consistent with the growing emphasis on human oversight and data validation in educational AI use^28^. Lastly, the Course & Institution Expectations factor underscores the importance of explicit guidelines and institutional clarity, reflecting a need for structured support and communication from universities and instructors^29^.

Few studies have attempted to measure AI literacy in the context of sport and exercise science education^30,31^. Existing tools primarily focus on general digital literacy or AI attitudes in broader higher education populations^32^. The present study extends this literature by situating AI competencies within the unique context of sport and exercise disciplines, where AI applications – such as performance analysis, motion tracking, and injury prediction—are increasingly integrated into teaching and professional practice.

The strong psychometric performance of this domain-specific tool suggests that contextualized measurement approaches may be useful for capturing AI-related competencies within applied disciplines^33^.

Moreover, the emphasis on Ethics & Disclosure and Trust & Verification resonates with recent studies highlighting ethical AI use and transparency as core competencies for future professionals^34^. The high loadings within these factors reflect students’ awareness of their responsibility to disclose AI use and verify outputs before integration into academic or professional contexts. These results suggest that students recognise the importance of ethical disclosure and verification when using AI tools. although practical implementation and institutional reinforcement remain essential. Although the measurement model demonstrated overall acceptable fit, the correlation between the Ethics & Disclosure (ED) and Trust & Verification (TV) constructs was relatively high (*r* = 0.85), approaching commonly suggested thresholds for discriminant validity. This strong association is theoretically plausible, as ethical disclosure of AI use in academic work often involves critically evaluating and verifying AI-generated information. Nevertheless, the two constructs capture conceptually distinct aspects of responsible AI engagement—ethical transparency in reporting AI use versus critical evaluation of AI outputs—and were therefore retained as separate dimensions in the measurement model.

The validated instrument provides educators and administrators with a robust tool to assess students’ AI-related competencies and perceptions. It can guide curriculum design by identifying areas where students may lack foundational AI understanding or ethical awareness, enabling targeted interventions^35^. The inclusion of the Course & Institution Expectations domain highlights a broader pedagogical implication: students desire clear institutional policies and practical training on AI use^36^. Universities should thus consider developing explicit guidelines, workshops, and learning modules that promote responsible AI integration aligned with disciplinary needs^37^.

From a broader perspective, this study contributes to advancing AI literacy frameworks in applied health and sport education. As AI tools become integral to professional decision-making, educators play a critical role in ensuring graduates possess both technical competence and ethical discernment^38^.

### Strengths and limitations

A key strength of this study lies in its methodological rigor, including the use of parallel analysis, polychoric correlation matrices, and split-sample validation to confirm the factor structure. The large sample size and high KMO value (0.95) indicate excellent sampling adequacy. Additionally, retaining items based on stringent psychometric criteria (e.g., primary loading ≥ 0.40, cross-loading < 0.30) ensured a parsimonious and theoretically coherent scale.

However, several limitations warrant consideration. First, the data were collected from sport and exercise science students at a specific set of institutions, which may limit generalizability to other disciplines or cultural contexts. In addition, the sample showed a notable gender imbalance, with approximately 80% of participants being male, which may affect the representativeness of the findings and the applicability of the instrument to more gender-balanced populations. Future research should therefore validate the instrument in more diverse samples and test its measurement invariance across demographic groups, educational levels, countries, and academic fields. Second, the study relied on self-reported responses, which may be subject to social desirability bias, particularly in domains involving ethics and disclosure. Finally, while internal consistency and construct validity were demonstrated, further research is needed to establish test-retest reliability and criterion validity against behavioural measures of AI engagement^39^.

Future studies could explore longitudinal applications of the tool to examine changes in AI literacy following targeted training or curriculum reform^40^. Additionally, expanding the framework to include affective and behavioural components, such as motivation to use AI or real-world usage patterns, may provide a more holistic understanding of students’ readiness for AI-integrated professional practice^41^.

## Conclusion

This study developed and validated a concise, reliable, and contextually grounded questionnaire to assess AI-related knowledge, ethical awareness, and expectations among sport and exercise science students. The instrument offers a practical means to evaluate and enhance AI literacy within sport education, supporting the responsible integration of emerging technologies into academic and professional practice.

## Supplementary Information

Below is the link to the electronic supplementary material.

Supplementary Material 1

Supplementary Material 2

## Data Availability

The dataset generated and analysed in this study contains confidential student information and cannot be publicly shared. De-identified data is available from the corresponding author upon reasonable request.
