# Supplementary material for: Development and validation of an AI use scale for sport and exercise science students
Source: Sci Rep. 2026 Mar 21;16:14467. doi: 10.1038/s41598-026-45316-4 (PMC13149980; doi:10.1038/s41598-026-45316-4)
Supplement: Supplementary file 1 — Supplementary Material 1 [file 41598_2026_45316_MOESM1_ESM.docx]

## ·Supplemental file 2

**Table S1 Initial Item Pool of the AI Use Scale for Sport and Exercise Science Students (16 Items)**

| **Module / Scale** | **Code** | **Item (English)** | **Response Format / Options** |
| --- | --- | --- | --- |
| Module A — AI Usage (not scored) | A1 | Have you ever used any AI tools? | Yes / No |
|  | A2 | How frequently do you use AI in everyday life? | Never / 1–2 times / 3–4 times / Almost every day / Multiple times per day |
|  | A3 | For which tasks do you mainly use AI? (select all that apply) | ① Coursework writing/summary ② Information search ③ Planning & scheduling ④ Communication & email ⑤ Data/code ⑥ Creative work (image/audio/video) ⑦ Training/rehabilitation-related ⑧ Other: ____ |
|  | A4 | Have you received any AI-related training/courses? | No / Yes, approximately __ hours |
| Subscale — AI Awareness (AW) | AW1 | I understand the definition of artificial intelligence. | 5-point Likert (1–5) |
|  | AW2 | I am familiar with some basic principles of AI (e.g., linear models, decision trees, machine learning). | 5-point Likert (1–5) |
|  | AW3 | I understand how AI ‘perceives’ the world (e.g., ‘seeing’, ‘hearing’) to complete tasks. | 5-point Likert (1–5) |
|  | AW4 | I can compare related AI concepts (e.g., the difference between deep learning and machine learning). | 5-point Likert (1–5) |
| Subscale — Ethics & Disclosure (ED) | B1 | When submitting assignments/reports/works, I state truthfully whether AI was used. | 5-point Likert (1–5) |
|  | B2 | When using AI in collaboration or in contexts involving others (e.g., teaching/coaching/tutoring), I inform them in advance. | 5-point Likert (1–5) |
|  | B3 | I understand and comply with my course/unit’s boundaries for AI use. | 5-point Likert (1–5) |
| Subscale — Trust & Verification (TV) | C1* | If AI provides no sources or evidence, I am less likely to adopt its outputs. | 5-point Likert (1–5) |
|  | C2 | If AI’s judgment conflicts with my own, a teacher/peer, or other sources, I am less likely to adopt it. | 5-point Likert (1–5) |
|  | C3* | I fact-check the content generated by AI. | 5-point Likert (1–5) |
|  | C4 | I can identify bias in AI-generated content. | 5-point Likert (1–5) |
|  | C5 | I maintain a skeptical or cautious attitude toward AI-generated content. | 5-point Likert (1–5) |
| Subscale — Course & Institution Expectations (CIE) | E1 | I want the university to offer systematic and practical AI literacy and hands-on courses. | 5-point Likert (1–5) |
|  | E2 | I want instructors to clearly specify which tasks may use AI, how to use it, and how to disclose it. | 5-point Likert (1–5) |
|  | E3 | I want the university to provide compliant and secure AI tools and accounts for staff and students (including privacy safeguards). | 5-point Likert (1–5) |
|  | E4 | I support restricting/prohibiting AI use or using alternative assessments in high‑stakes exams, provided the rules are clear. | 5-point Likert (1–5) |

**Note**: * Items **C1, and C3** were removed during exploratory factor analysis because they did not meet the predefined item retention criteria (**primary loading ≥ 0.40, largest cross-loading < 0.30, and communality ≥ 0.30**). The final validated scale therefore includes **14 scored items**.

**Table S2 Final Validated Version of the AI Use Scale for Sport and Exercise Science Students (14 Items)**

| **Module / Scale** | **Code** | **Item (English)** | **Response Format / Options** |
| --- | --- | --- | --- |
| Module A — AI Usage (not scored) | A1 | Have you ever used any AI tools? | Yes / No |
|  | A2 | How frequently do you use AI in everyday life? | Never / 1–2 times / 3–4 times / Almost every day / Multiple times per day |
|  | A3 | For which tasks do you mainly use AI? (select all that apply) | ① Coursework writing/summary ② Information search ③ Planning & scheduling ④ Communication & email ⑤ Data/code ⑥ Creative work (image/audio/video) ⑦ Training/rehabilitation-related ⑧ Other: ____ |
|  | A4 | Have you received any AI-related training/courses? | No / Yes, approximately __ hours |
| Subscale — AI Awareness (AW) | AW1 | I understand the definition of artificial intelligence. | 5-point Likert (1–5) |
|  | AW2 | I am familiar with some basic principles of AI (e.g., linear models, decision trees, machine learning). | 5-point Likert (1–5) |
|  | AW3 | I understand how AI ‘perceives’ the world (e.g., ‘seeing’, ‘hearing’) to complete tasks. | 5-point Likert (1–5) |
|  | AW4 | I can compare related AI concepts (e.g., the difference between deep learning and machine learning). | 5-point Likert (1–5) |
| Subscale — Ethics & Disclosure (ED) | B1 | When submitting assignments/reports/works, I state truthfully whether AI was used. | 5-point Likert (1–5) |
|  | B2 | When using AI in collaboration or in contexts involving others (e.g., teaching/coaching/tutoring), I inform them in advance. | 5-point Likert (1–5) |
|  | B3 | I understand and comply with my course/unit’s boundaries for AI use. | 5-point Likert (1–5) |
| Subscale — Trust & Verification (TV) | C2 | If AI’s judgment conflicts with my own, a teacher/peer, or other sources, I am less likely to adopt it. | 5-point Likert (1–5) |
|  | C4 | I can identify bias in AI-generated content. | 5-point Likert (1–5) |
|  | C5 | I maintain a skeptical or cautious attitude toward AI-generated content. | 5-point Likert (1–5) |
| Subscale — Course & Institution Expectations (CIE) | E1 | I want the university to offer systematic and practical AI literacy and hands-on courses. | 5-point Likert (1–5) |
|  | E2 | I want instructors to clearly specify which tasks may use AI, how to use it, and how to disclose it. | 5-point Likert (1–5) |
|  | E3 | I want the university to provide compliant and secure AI tools and accounts for staff and students (including privacy safeguards). | 5-point Likert (1–5) |
|  | E4 | I support restricting/prohibiting AI use or using alternative assessments in high‑stakes exams, provided the rules are clear. | 5-point Likert (1–5) |
