# Supplementary material for: Development and validation of an AI use scale for sport and exercise science students
Source: Sci Rep. 2026 Mar 21;16:14467. doi: 10.1038/s41598-026-45316-4 (PMC13149980; doi:10.1038/s41598-026-45316-4)
Supplement: Supplementary file 2 — Supplementary Material 2 [file 41598_2026_45316_MOESM2_ESM.docx]

**Supplemental file 1**

**Table S1. Item-level KMO (MSA) Ranking**

| **Item** | **QC3** | **QB1** | **QD3** | **QB2** | **QC4** | **QC5** | **QD2** | **QE1** | **QE2** | **QE4** | **QB3** | **QAW1** | **QC1** | **QE3** | **QAW4** | **QC2** | **QAW2** | **QAW3** | **QD1** |
| --- | --- | --- | --- | --- | --- | --- | --- | --- | --- | --- | --- | --- | --- | --- | --- | --- | --- | --- | --- |
| MSA | 0.98 | 0.97 | 0.97 | 0.96 | 0.96 | 0.96 | 0.96 | 0.96 | 0.96 | 0.96 | 0.95 | 0.94 | 0.94 | 0.94 | 0.92 | 0.92 | 0.91 | 0.89 | 0.84 |

**Table S2. Parallel Analysis (PA) Results**

| **Component** | **FA eigen (observed)** | **FA eigen (random mean)** | **Retain (FA by PA)** | **PC eigen (observed)** |
| --- | --- | --- | --- | --- |
| 1 | 9.7947 | 0.3688 | Yes | 10.1552 |
| 2 | 1.2756 | 0.2015 | Yes | 1.8208 |
| 3 | 0.4538 | 0.1647 | Yes | 0.7891 |
| 4 | 0.2156 | 0.1321 | Yes | 0.5599 |
| 5 | 0.0772 | 0.1021 | No | 0.4509 |
| 6 | 0.0010 | 0.0750 | No | 0.3771 |
| 7 | -0.0331 | 0.0486 | No | 0.3215 |
| 8 | -0.1023 | 0.0237 | No | 0.2586 |
| 9 | -0.1207 | -0.0007 | No | 0.2280 |
| 10 | -0.1357 | -0.0262 | No | 0.2093 |
| 11 | -0.1671 | -0.0511 | No | 0.1960 |
| 12 | -0.1807 | -0.0762 | No | 0.1481 |
| 13 | -0.2027 | -0.1031 | No | 0.1429 |
| 14 | -0.2252 | -0.1309 | No | 0.1269 |
| 15 | -0.3740 | -0.1612 | No | 0.1140 |
| 16 | -0.4818 | -0.1983 | No | 0.1017 |

Note: PA = Parallel Analysis. Retention is based on FA observed > FA random mean.

**Table S3 Pre-trimmed EFA results**

| Item | **AI Awareness** | **Ethics & Disclosure** | **Trust & Verification** | **Course & Institution Expectations** | h2 |
| --- | --- | --- | --- | --- | --- |
| QAW1 | 0.53 |  |  |  | 0.53 |
| QAW2 | 0.85 |  |  |  | 0.77 |
| QAW3 | 0.93 |  |  |  | 0.84 |
| QAW4 | 0.87 |  |  |  | 0.81 |
| QB1 |  | 0.6 |  |  | 0.71 |
| QB2 |  | 0.71 |  |  | 0.79 |
| QB3 |  | 0.68 |  |  | 0.85 |
| QC1 |  | 0.33 | 0.52 |  | 0.76 |
| QC2 |  |  | 0.95 |  | 0.82 |
| QC3 |  |  | 0.46 | 0.33 | 0.75 |
| QC4 |  |  | 0.75 |  | 0.81 |
| QC5 |  |  | 0.61 |  | 0.81 |
| QE1 |  |  |  | 0.8 | 0.71 |
| QE2 |  |  |  | 0.92 | 0.93 |
| QE3 |  |  |  | 0.89 | 0.87 |
| QE4 |  |  |  | 0.57 | 0.7 |
| Eigenvalues | 3.05 | 2.44 | 3.33 | 3.67 |  |
| Variance explained (%) | 24.40% | 19.56% | 26.69% | 29.35% |  |

**Table S4. Item standardized loadings in CFA, with 95% CIs and R²**

| **Factor** | **Item** | **Std. loading** | **95% CI** | **p** | **R²** |
| --- | --- | --- | --- | --- | --- |
| **AI Awareness (AW)** | QAW1 | 0.88 | (0.84, 0.91) | < .001 | 0.77 |
|  | QAW2 | 0.88 | (0.86, 0.91) | < .001 | 0.78 |
|  | QAW3 | 0.86 | (0.83, 0.89) | < .001 | 0.74 |
|  | QAW4 | 0.90 | (0.88, 0.93) | < .001 | 0.82 |
| **Ethics & Disclosure (ED)** | QB1 | 0.91 | (0.89, 0.93) | < .001 | 0.83 |
|  | QB2 | 0.93 | (0.91, 0.94) | < .001 | 0.86 |
|  | QB3 | 0.96 | (0.94, 0.97) | < .001 | 0.91 |
| **Trust & Verification (TV)** | QC2 | 0.78 | (0.75, 0.82) | < .001 | 0.62 |
|  | QC4 | 0.89 | (0.86, 0.92) | < .001 | 0.79 |
|  | QC5 | 0.95 | (0.93, 0.97) | < .001 | 0.90 |
| **Course & Institution Expectations (CIE)** | QE1 | 0.89 | (0.87, 0.92) | < .001 | 0.79 |
|  | QE2 | 0.87 | (0.84, 0.90) | < .001 | 0.76 |
|  | QE3 | 0.97 | (0.96, 0.98) | < .001 | 0.93 |
|  | QE4 | 0.93 | (0.91, 0.95) | < .001 | 0.86 |

**Table S5. latent correlations**

| **Row factor** | **Column factor** | **latent correlations** |
| --- | --- | --- |
| AW | ED | 0.70 |
| AW | CIE | 0.53 |
| AW | TV | 0.60 |
| TV | CIE | 0.82 |
| ED | TV | 0.85 |
| ED | CIE | 0.76 |

**Note.** Φ = observed first-order latent factor correlation; AW = AI Awareness; ED = Ethics & Disclosure; TV = Trust & Verification; CIE = Course & Institution Expectations.
